# Re-Evaluating the Effects of Obesity on Cancer Immunotherapy Outcomes in Renal Cancer: What Do We Really Know?

**DOI:** 10.3389/fimmu.2021.668494

**Published:** 2021-08-05

**Authors:** Kristine I. Farag, Amani Makkouk, Lyse A. Norian

**Affiliations:** ^1^Science and Technology Honors Program, University of Alabama at Birmingham, Birmingham, AL, United States; ^2^Department of Pharmacology, Adicet Bio, Menlo Park, CA, United States; ^3^Department of Nutrition Sciences, University of Alabama at Birmingham, Birmingham, AL, United States; ^4^O’Neal Comprehensive Cancer Center, University of Alabama at Birmingham, Birmingham, AL, United States; ^5^Nutrition Obesity Research Center, University of Alabama at Birmingham, Birmingham, AL, United States

**Keywords:** cancer, immune checkpoint blockade, immunotherapy, obesity, renal cancer, anti-tumor immunity

## Abstract

Obesity has reached global epidemic proportions and its effects on interactions between the immune system and malignancies, particularly as related to cancer immunotherapy outcomes, have come under increasing scrutiny. Although the vast majority of pre-clinical murine studies suggest that host obesity should have detrimental effects on anti-tumor immunity and cancer immunotherapy outcomes, the opposite has been found in multiple retrospective human studies. As a result, acceptance of the “obesity paradox” paradigm, wherein obesity increases cancer risk but then improves patient outcomes, has become widespread. However, results to the contrary do exist and the biological mechanisms that promote beneficial obesity-associated outcomes remain unclear. Here, we highlight discrepancies in the literature regarding the obesity paradox for cancer immunotherapy outcomes, with a particular focus on renal cancer. We also discuss multiple factors that may impact research findings and warrant renewed research attention in future studies. We propose that specific cancer patient populations may be affected in fundamentally different ways by host obesity, leading to divergent effects on anti-tumor immunity and/or immunotherapy outcomes. Continued, thoughtful analysis of this critical issue is therefore needed to permit a more nuanced understanding of the complex effects of host obesity on cancer immunotherapy outcomes in patients with renal cancer or other malignancies.

## Introduction

Obesity is a multi-faceted disease that is linked to an increased risk of developing heart disease, diabetes mellitus, hypertension, and 13 types of cancer, including renal cell carcinoma (RCC) (1, 2). The body mass index (“BMI”, defined as a person’s weight in kg divided by height in m^2^) is a proxy for overall body fatness and is the most frequently used method of defining obesity (*i.e.* when an individual’s BMI is ≥ 30 kg/m^2^, according to World Health Organization classifications) (3). In the United States, it is estimated that approximately 40% of the adult population has BMI-defined obesity (3), a percentage predicted to increase to 50% by 2050 (4). Despite its ease of use, BMI fails to capture the range of metabolic and physiologic perturbations that can accompany adult obesity and therefore does not reflect the heterogeneity of this disease. As obesity approaches epidemic proportions globally, researchers continue to investigate the mechanisms by which it induces immunological dysfunction and impacts cancer progression and treatment efficacy. Here, we will summarize the known effects of obesity on anti-tumor immunity and cancer immunotherapy outcomes, particularly as related to renal cancer, and will highlight areas of discrepancy that we believe require additional investigation.

### Obesity Is a Heterogeneous Disease

Before discussing the effects of obesity on anti-tumor immunity or cancer immunotherapy outcomes, it is important to understand something of the complex and heterogeneous nature of this disease. Although it is widely recognized that insulin resistance, inflammation, and dyslipidemia (i.e., abnormally high levels of lipids in circulation) are hallmarks of obesity, these traits are not present to the same extent in all afflicted individuals. As mentioned above, ~40% of adults in the U.S. currently have obesity (3). In contrast, the Centers for Disease Control estimated that Type 2 Diabetes, defined in part by a state of acquired resistance to insulin, affected approximately 34.1 million U.S. adults in 2018, for a percentage of 13% (https://www.cdc.gov/diabetes/pdfs/data/statistics/national-diabetes-statistics-report.pdf). Thus, calculations based on these numbers indicate that fewer than half of all adults with obesity have clinically diagnosed Type 2 Diabetes, although many more likely have undiagnosed pre-diabetes. Obesity-associated inflammation is also not ubiquitous. For example, C-reactive protein (CRP) is an important clinical indicator of inflammation, but studies have found that elevated CRP occurs in only 30-60% of adults with obesity (5, 6). Furthermore, the concept of “metabolically healthy obesity” has received much attention in recent years (7, 8). Individuals with metabolically healthy obesity are defined as having low or absent dyslipidemia, hypertension, and insulin resistance *versus* individuals with metabolically unhealthy obesity who display these conditions (7, 8). Although the prevalence of metabolically healthy *versus* unhealthy obesity remains an area of debate, it is clear that the physiologic complications associated with obesity can diverge widely between individuals. Body composition also varies with obesity; some adults have high visceral adiposity, whereas others have higher subcutaneous adiposity, and still others have a loss of lean muscle also referred to as sarcopenia or myopenia. Research studies that use BMI as the sole obesity-defining metric do not capture this heterogeneity, a deficiency that is beginning to be addressed more frequently by the research community. The potential roles of obesity-associated inflammation and body composition will be discussed below, as there is mounting evidence that such factors influence the efficacy of immune checkpoint inhibitors (ICIs) in advanced cancer patients.

## Obesity-Associated Changes in Anti-Tumor Immunity

### Pre-Clinical Murine Findings Reveal Multiple Breakdowns in Immune Function

**Figure 1:**
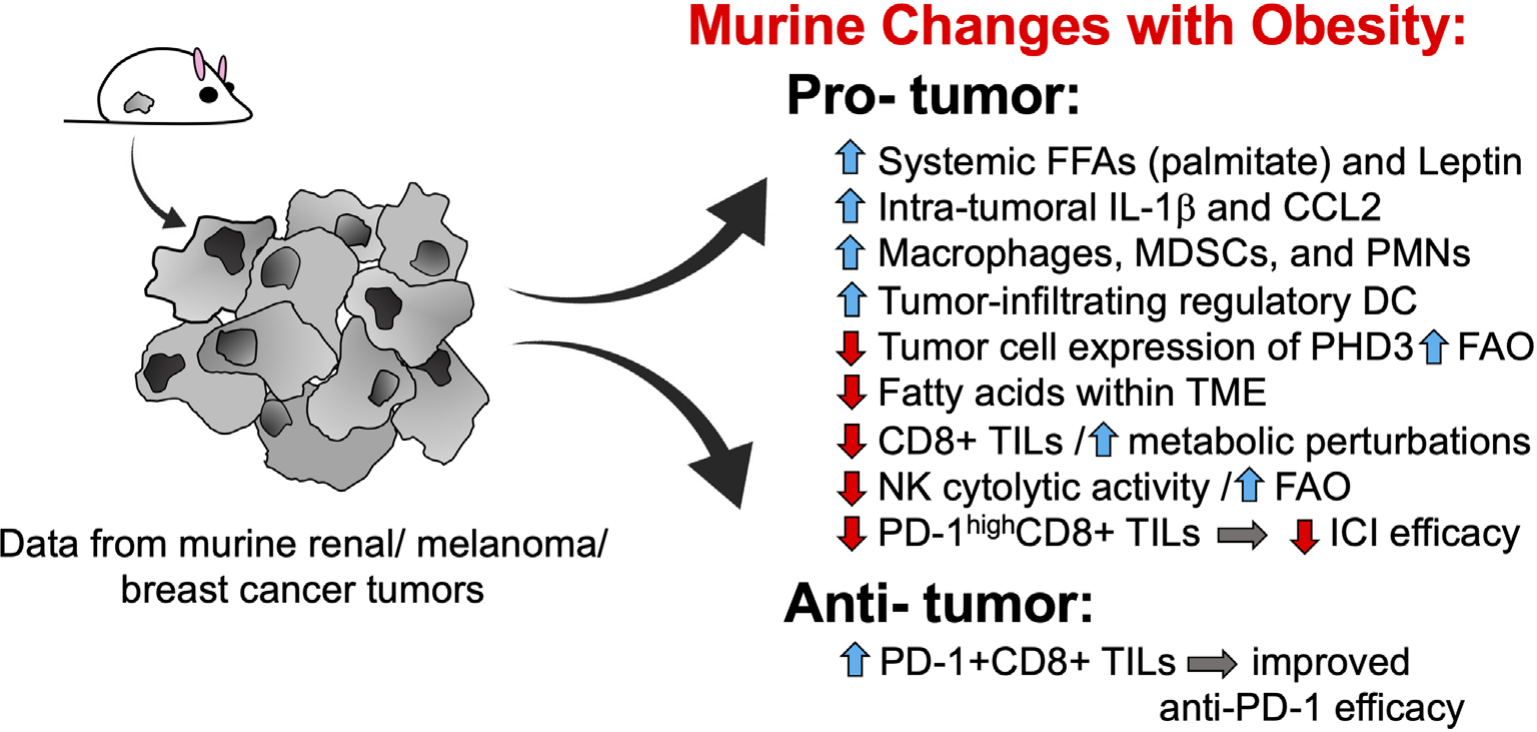
Murine models reveal multiple pro-tumorigenic changes to anti-tumor immunity with host obesity. Pre-clinical tumor modeling provides evidence that host obesity is associated with a net increase in pro-tumorigenic changes to immune responses, although obesity-associated alterations that favor tumor clearance and/or enhanced ICI efficacy have been described. DCs, dendritic cells; FAO, fatty acid oxidation; FFAs, free fatty acids; ICI, immune checkpoint inhibitor; IL-1, interleukin-1; MDSCs, myeloid-derived suppressor cells; NK, natural killer; PD-1, programmed cell death-1; PHD3, prolylhydroxylase-3; PMNs, polymorphonuclear cells a.k.a. neutrophils; TILs, tumor-infiltrating lymphocytes; TME, tumor microenvironment.

Almost without fail, pre-clinical studies on obesity – in the presence or absence of tumor growth – have reported detrimental effects on immune function in general and anti-tumor immunity, in particular. For example, murine studies in tumor-free mice have found that obesity induces lymph node atrophy (9), impedes lymphatic transport (10), and reduces T cell receptor (TCR) diversity (11). Although obesity was found to increase the relative frequency of conventional dendritic cells in the spleens of tumor-free mice, splenic dendritic cells from obese animals had a reduced stimulatory capacity, resulting in weaker antigen-specific CD8+ T cell proliferation (12). In mice with solid tumors, host obesity has been found to increase the accumulation of tumor-promoting myeloid cells, including macrophages (13), neutrophils (14) and myeloid-derived suppressor cells (MDSCs) (15, 16) within the tumor microenvironment (**Figure 1**). In obese mice with mammary tumors, intratumoral MDSCs expressing FasL are able to trigger heightened levels of Fas-mediated apoptosis in activated CD8+ tumor-infiltrating lymphocytes (TILs) resulting in a depletion of effector T cells relative to what is seen in lean mice (17). In pre-clinical renal cancer modeling, aggressive intratumoral MDSC infiltration in mice with diet-induced obesity (DIO) is facilitated by elevated local concentrations of IL-1b and CCL2, resulting in unfavorable ratios of MDSCs to CD8+ TILs (18). Host obesity was also found to cause renal tumor-infiltrating dendritic cells to acquire suppressive capacities, resulting in inhibition of CD8+ T cell proliferation (19). Thus, findings from pre-clinical murine models indicate that obesity alters normal immune function in ways that culminate in a net impairment of protective anti-tumor immunity (**Figure 1**).

Not surprisingly, obesity has also been linked to metabolic perturbations in key leukocyte subsets that impair their cytolytic activity and ability to control tumor progression. As we reviewed this topic in depth in 2020 (20), only the most recent advances will be highlighted here. Using a murine model of DIO, Michelet et al. reported that host obesity induced a robust transcriptomic remodeling of NK cells in tumor-free mice (21) that led to altered peroxisome proliferator-activated receptor (PPAR) signaling and dysregulated cellular metabolism, culminating in increased lipid accumulation within NK cells and decreased cytolytic activity. When mice with DIO were challenged with melanoma tumors, NK cells were no longer able to counteract tumors, resulting in more rapid tumor outgrowth (21). Of note, NK metabolic and functional defects in mice with DIO were linked specifically to cellular uptake of free fatty acids such as palmitate and could be reversed by blocking fatty acid oxidation with etomoxir, a drug that inhibits carnitine palmitoyltransferase 1-b (Cpt1b), a key enzyme in the fatty acid oxidation metabolic pathway. In agreement with these findings, two groups subsequently reported obesity-associated metabolic defects in CD8+ TILs. Zhang et al. found that spontaneous mammary tumors in mice with DIO displayed more aggressive outgrowth (22). Accelerated tumor growth was accompanied by metabolic perturbations in CD8+ TILs that impaired their anti-tumor activity, including elevated STAT3 signaling, increased Cpt1b expression, and increased fatty acid oxidation at the expense of glycolysis (23). Notably, obesity-associated leptin was found to induce STAT3 signaling in CD8+ TILs. Blocking leptin, STAT3 signaling, or fatty acid oxidation with the Cpt1 inhibitor etomoxir restored CD8+ TIL effector function and slowed tumor growth, with the latter finding reflecting the observations of Michelet et al. in their NK study. In late 2020, Ringel et al. published findings that solidified the negative effects of obesity on CD8 TIL metabolism and function (24). These authors found that melanoma tumor cells responded to host obesity by increasing their uptake of free fatty acids and shifting their metabolism to elevate fatty acid oxidation; the result was a depletion of fatty acids locally within the tumor microenvironment and more rapid tumor outgrowth (24) (**Figure 1**). In contrast, CD8+ TIL from melanoma tumors did not exhibit this type of metabolic plasticity and consequently demonstrated a loss of proliferation and Granzyme B production, reflecting their diminished capacity for tumor control. Blocking tumor cell fatty acid metabolism by genetic overexpression of prolyl hydroxylase-3 (PHD3), an enzyme that normally represses fatty acid oxidation but is down-regulated in tumor cells from DIO mice, restored CD8+ TIL function and slowed tumor outgrowth. Notably, evidence for the intra-tumoral down-regulation of PHD3 was found in colon adenocarcinoma patients with obesity. Reduced expression of PHD3 was also identified in immunologically “cold” tumors (*i.e.* those with low CD8+ T cell signatures) from patients with colon adenocarcinoma or clear cell renal cell carcinoma, but not melanoma (24). This finding is critical, as it suggests that obesity-linked metabolic perturbations differ between tumor types, illustrating the need to assess the effects of obesity on anti-tumor immunity and immunotherapy outcomes on a tumor-by-tumor basis.

Host obesity has also been found to promote CD8+ TIL exhaustion *via* the effects of leptin. A 2018 study by Wang et al. reported that leptin can increase programmed cell death 1 (PD-1) expression on CD8+ T cells (25). Because obesity is tightly linked to elevated leptin expression in mice, it was therefore not surprising that the authors found obesity to be associated with higher frequencies of PD-1+CD8+ TILs in melanoma tumors (25). However, despite the severe functional exhaustion of CD8+ TILs from mice with DIO, obese animals actually exhibited a better response to anti-PD-1 therapy, as evidenced by larger reductions in tumor volumes *versus* outcomes in lean control (25) (**Figure 1**). These findings led the authors to conclude that obesity-associated increases in leptin resulted in more PD-1 target being expressed on CD8+ TILs, which in turn led to enhanced anti-PD-1 efficacy.

However, results from several other murine studies indicate that such beneficial effects of DIO on immunotherapeutic efficacy are not universal. For example, Murphy et al. reported that anti-CTLA-4 monotherapy was less effective in renal tumor-bearing mice with DIO than in lean mice (26). More recently, we determined that DIO was associated with a greater percentage of mice that failed to respond to a combinatorial immunotherapy consisting of an *in situ* T cell priming agent administered upstream of anti-PD-1, despite the fact that intra-renal tumor burdens were equivalent in DIO and lean mice at the time of treatment initiation (18). Immunogenetic analysis of excised renal tumors in our study revealed that the immune signatures of treatment responders from both obese and lean mice were comparable and displayed an extensive remodeling of the tumor microenvironment in response to therapy administration. This remodeling was absent in obese non-responders, whose tumor immune signatures were similar to those of treatment-naive mice. Specifically, obese non-responders exhibited a weak CD8+ T cell signature coupled with a strong myeloid cell signature - findings that were confirmed at the cellular level and were linked to elevated intratumoral IL-1β concentrations in obese animals. Notably, evaluations of obese-resistant mice that had been fed the same high fat diet for 20 weeks illustrated that host obesity, and not the high fat diet or its components, was the cause of diminished immunotherapeutic efficacy, because obese-resistant mice responded as well to this combination therapy as did control mice on a low-fat chow diet (18). More recently, we found that obesity-associated defects in T cell responses extended to the intratumoral CD4+ compartment and CD8+ T cells in tumor-draining lymph nodes in mice with renal tumors that were treated with combinatorial anti-CTLA-4 (27). In 2021, Kheum et al. reported that a combinatorial immunotherapy consisting of anti-PD-1/anti-CTLA-4/anti-LAG-3 was less effective in obese mice fed a Western Diet than in age-matched lean controls on standard diet in both the B16 melanoma and MC38 colon carcinoma models (28). It is notable that further investigation by these authors determined that diminished immunotherapeutic efficacy was not due to elevated body weight but was instead caused by high levels of fructose in the administered Western Diet, which caused tumor cells to upregulate expression of the protein hemeoxygenase-1 (HO-1), thereby protecting them from T cell-mediated killing (28). The above disparities in murine findings indicate that the effects of host obesity are not easily summarized as being either only detrimental or only beneficial. More research is urgently needed to determine whether the beneficial effects of obesity are dependent upon the type of immunotherapy or obesogenic diet being administered, the type and anatomic location of the tumor model in question, or the presence of obesity-associated factors such as IL-1β or other inflammatory mediators that may be expressed to varying degrees between one individual mouse or strain *versus* another.

### Obesity-Linked Alterations to Human Immunity in the Absence and Presence of Renal Cancer

In humans, the effects of obesity on immune composition and function have been examined in several recent studies, but consistent trends remain far less obvious than those identified in mice. Elisia et al. studied the effects of obesity on leukocyte composition and T cell function in tumor-free individuals. These authors reported that obesity was associated with heightened systemic inflammation, as noted by increases in plasma IL-6, IL-17, C-reactive protein (CRP), and prostaglandin E2 (PGE2) (29). In donors with obesity, stimulation of PBMCs with Herpes Simplex Virus 1 (HSV-1) led to elevated concentrations of the pro-angiogenic vascular endothelial growth factor (VEGF). Furthermore, anti-CD3/anti-CD28 stimulation of T cells in bulk peripheral blood mononuclear cell (PBMC) preparations resulted in heightened interferon γ (IFNγ) secretion in donors with obesity, despite the fact that fewer CD8+ T cells and CD56+ NK cells were present (29). These findings suggest that in humans, obesity may be associated with hyper-activation of cytolytic cells. In contrast, Wang et al. determined that in rhesus macaques and humans with obesity (the latter defined as BMI ≥ 30 kg/m^2^), increased frequencies of PD-1+ T cells were present in the peripheral blood. These PD-1+ cells had reduced proliferative capacity, illustrating a dysfunctional state. In tumor-free human donors with obesity, the authors found that increasing leptin concentrations in the blood were positively associated with increased frequencies of PD-1+CD8+ T cells, which aligned with their murine data showing that leptin promoted increased PD-1 expression on CD8+ T cells (25).

**Figure 2:**
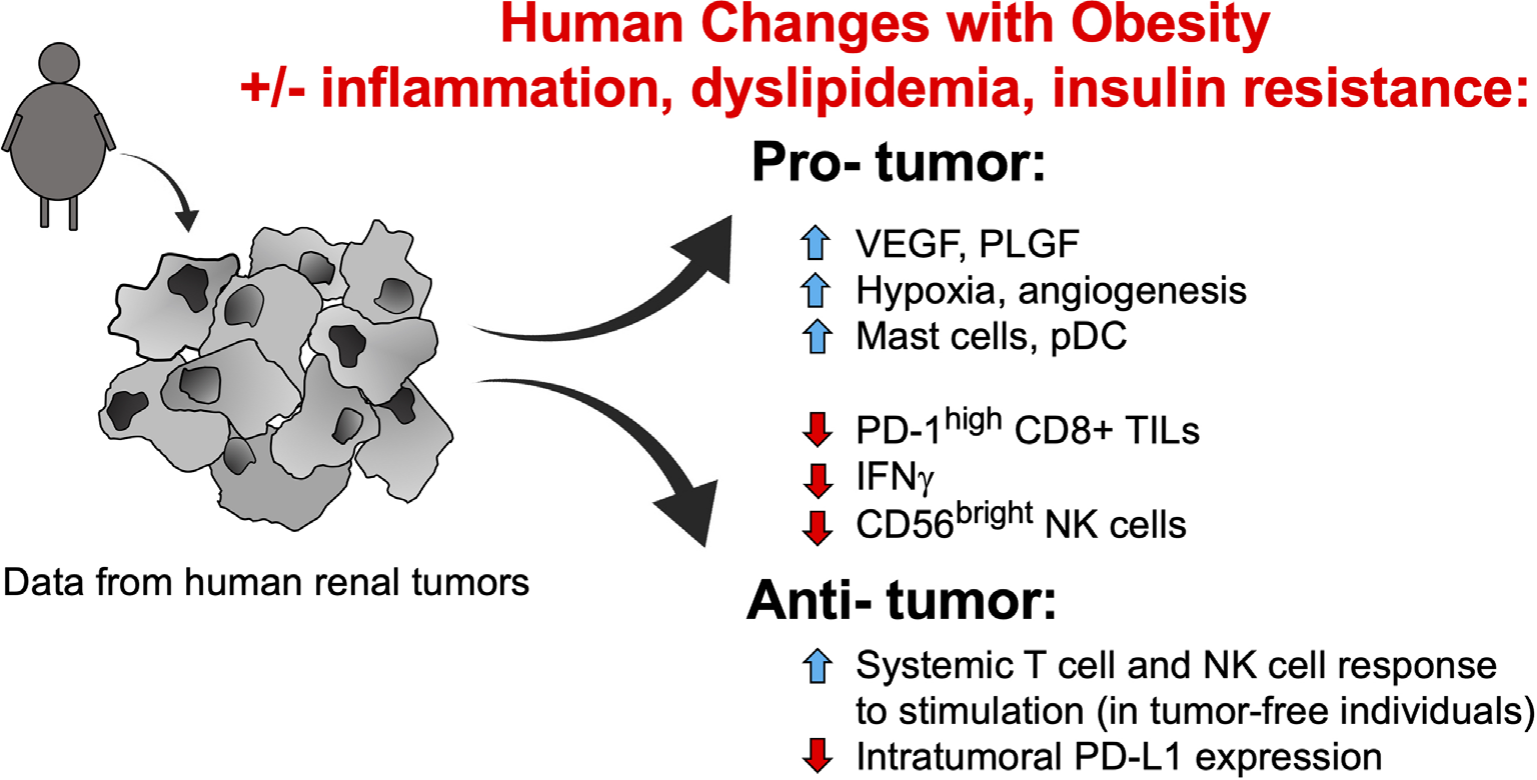
Identified obesity-associated changes in humans that are predicted to facilitate renal tumor progression. Studies of tumor-free individuals and human renal tumors suggest that many obesity-associated changes to the immune compartment and soluble growth factors should favor tumor progression, although some alterations that would promote enhanced tumor clearance and/or ICI efficacy have also been noted. Note that the contributions of obesity-associated factors such as inflammation, dyslipidemia, and insulin resistance remain unclear, in terms of their combined effects on immune function. NK, natural killer; PD-1, programmed cell death-1; PD-L1, programmed cell death ligand 1; pDCs, plasmacytoid dendritic cells; PLGF, placental growth factor; TILs, tumor-infiltrating lymphocytes; VEGF, vascular endothelial growth factor.

At this time, however, the connections between obesity, circulating leptin concentrations, and PD-1 expression remain unclear, particularly in cancer patients. Two recent studies did not observe positive associations between leptin concentration and the frequency of PD-1+CD8+ T cells. Khojandi et al. found no significant associations (positive or negative) between either BMI or plasma leptin concentrations and PD-1 expression on peripheral blood CD8+ T cells in a cohort of 27 melanoma patients, 11 breast cancer patients, and 30 non-Hodgkin lymphoma patients who ranged from having a normal body weight to having obesity (30). Our own examination of treatment-naive renal cancer patients found that higher plasma leptin concentrations were associated with reduced frequencies of peripheral blood PD-1+CD8+ T cells; in this patient cohort higher BMIs were also associated with reduced frequencies of activated CD45RO+CD8+ T cells (31). We found that in the renal tumor microenvironment, pro-angiogenic factors VEGF-A and placental growth factor (PLGF) were elevated in subjects with obesity (31), whereas the frequency of activated PD-1^high^CD8+ T cells was reduced (18) (**Figure 2**). However, our nanoString analysis of immune-related genes in treatment-naive human renal tumors revealed that of the ~750 genes examined, surprisingly few were altered by obesity (31). Similar conclusions were reached by Sanchez et al., who performed an unbiased transcriptomic analysis of renal tumors from both treatment-naive patients and those treated with tyrosine kinase inhibitors (TKIs) (32). The authors found that in patients who had received TKI therapy, obesity was associated with increased hypoxia and angiogenesis in tumors, whereas tumor infiltration by total leukocytes, T cells, and myeloid cells was unchanged (32). These same tumors displayed an increased frequency of plasmacytoid dendritic cells but decreased expression of IFNg and PD-ligand 1 (PD-L1) (**Figure 2**). In renal tumors from treatment-naive individuals associated with The Cancer Genome Atlas project, the authors’ transcriptomic analysis suggested an increase in mast cells but a decrease in CD56^bright^ NK cells (32), with the latter finding reminiscent of the Elisia et al. study (29). The mast cell finding is intriguing, given the fact that mast cell infiltration of tumors was recently identified as a mechanism of resistance to anti-PD-1 therapy in humanized mice with melanoma tumors (33). If mast cells in renal tumors exert similar functions, the Sanchez finding would suggest that obesity should be detrimental to anti-PD-1 outcomes in RCC patients – a controversial idea that we discuss further below. One caveat to these transcriptomic data is that flow cytometric analysis of a second cohort of treatment-naive renal tumors (n = 7 normal weight RCC patients and n= 16 RCC patients with obesity) by Sanchez et al. identified no changes in the frequency of any leukocyte population examined (i.e. CD4 T cells, CD8 T cells, B cells, Tregs, neutrophils, dendritic cells), although mast cells were unfortunately not analyzed (32). Thus, the Sanchez results are important because they illuminate the heterogeneity present within renal tumors from subjects with obesity, as their findings varied across patient cohorts. To date, obesity has been linked to increased angiogenesis and decreased PD-1 or PD-L1 expression on CD8+ T cells in more than one study, so these may represent common obesity-associated characteristics of the human renal tumor environment. Many other aspects of the intratumoral leukocyte response either appear to be unchanged by obesity or to exhibit variability across study cohorts. Clearly, more work is needed before a cohesive picture emerges regarding the nature and magnitude of obesity-associated changes in leukocyte composition within human renal tumors.

## Re-Examining the Obesity Paradox Paradigm in Cancer Immunotherapy

Although most pre-clinical studies predict that obesity’s impact on the immune system should potentiate tumor proliferation and outgrowth, and obesity has been linked to an increased prevalence of renal and other cancers, multiple retrospective studies of cancer patients have found that obesity has beneficial effects on cancer immunotherapy outcomes. This dichotomy between obesity increasing cancer risk but later improving cancer immunotherapy outcomes is referred to as the “obesity paradox”. Even though the obesity paradox is frequently viewed as a generalization that applies to all or most cancer immunotherapy outcomes, mounting evidence to the contrary does exist and should be examined for the biological insights it can provide. Here, we emphasize areas of divergent results, with a particular focus on renal cancer, to summarize outcomes and discuss confounding factors, such as gender, fat distribution, and immunotherapy type or dose, that may impact observed data trends. Continued investigation into this critical area of research is needed so that optimal patient care decisions can be made.

### Evidence in Support of the Beneficial Effects of Obesity

#### Studies in Melanoma and Tumor Types Other Than Renal Cancer

Positive associations between obesity, as defined by BMI, and increased responsiveness to immune checkpoint inhibitor (ICI) therapies (i.e., antibodies blocking the interaction of PD-1 with its ligand PD-L1, or the interaction of CTLA-4 with its ligands B7-1 and B7-2) have been observed in cancer patients with varying tumor types. In one of the first of such studies, McQuade et al. evaluated progression-free survival (PFS) and overall survival (OS) in a retrospective study of six independent patient cohorts with metastatic melanoma, two of which contained patients treated with immunotherapies, two with targeted therapies, and two with chemotherapy (34). The immunotherapies used were any of the following ICI agents: anti-PD-1 (nivolumab, pembrolizumab), anti-PD-L1 (atezolizumab), or anti-CTLA-4 (ipilimumab) + dacarbazine (chemotherapy). The authors found that survival benefits associated with obesity were present in men treated with ICI or targeted therapies, corresponding with significantly lower hazard ratios (HR) for death (targeted therapies HR for men = 0.51[95% CI, 0.34-0.76] (35, 36); immunotherapies HR for men = 0.55 [95% CI, 0.32-0.93]; chemotherapies for men = 1.81 [95% CI, 0.71-1.97]) (**Table 1**). Notably, these beneficial associations between obesity and ICI outcomes were only significant in male patients with melanoma (34). In women with melanoma, obesity provided no benefit for either progression free survival (PFS) (immunotherapies HR for women = 1.11 [95% CI, 0.72-1.72]); or overall survival (OS) (immunotherapies HR for women = 0.90 [95% CI, 0.54-1.50]) (**Table 2**), for reasons that have remained elusive.

**Table 1:** Studies supporting the obesity paradox for cancer immunotherapy outcomes.

First author (Year)	Tumor type	Cohort size	Trial name or SOC	Obesity metric used	ICI used	Controlled prognostic factors	Outcomes
McQuade (34)	melanoma	213 males	retrospective analyses from 4 US and Australian centers	BMI: per WHO for NW, OVWT, OB.	anti-PD-1, anti-PD-L1, or anti-CTLA-4 + dacarbazine (chemotherapy)	age, AJCC & disease stage, LDH status, ECOG status	OB *vs* NW: males have improved PFS and OS
				UWT excluded			
Donnelly (37)	melanoma	423	prospective NYU Interdisciplinary Melanoma Cooperative Group	BMI: per WHO standards for UW, NW, OVWT, OB	anti-PD-1, anti-CTLA-4, or combination	1st *versus* 2nd+ line therapy; age, gender, tumor stage, LDH, ECOG, # metastatic sites	OB and OVWT: 1st line ICI trended toward improved PFS and OS.
							OB and OVWT with combo Tx: improved PFS and trend toward improved OS
Khojandi (30)	melanoma	129	trial NCT00094653	BMI: OB *vs* OVWT+NW+UWT	anti-CTLA-4	sex	OB improved OS
Labadie (38)	RCC	90	Mix of trials and retrospective	BMI: per WHO standards for UW, NW, OVWT, OB	mixed anti-PD-1/PD-L1	primary resistance *versus* primary response to ICI	Primary response in 58%: improved PFS with increasing BMI and irAE occurrence
Sanchez (32)	RCC	129	MSK Observational Immunotherapy Cohort	BMI: per WHO standards for OB *vs* NW	mixed: anti-PD-1, anti-PD-L1, anti-PD-1+anti-CTLA-4, anti-PD-1+anti-PD-L1	age, sex, IMDC score	OB: trend toward improved OS (not sig.). Unadjusted analysis without IMDC risk score showed OB was beneficial.
Kichenadasse (35)	NSCLC	1434	clinical trials: NCT02008227, NCT01903993, NCT02031458, NCT01846416	BMI: As per WHO for NW, OVWT, OB.	anti-PD-L1	age, sex, race, ECOG, smoking status, tumor subtype, # tumor sites, PD-L1 expression, LDH, CRP, NLR	OB *vs* NW: improved OS; especially for PD-L1+ tumors; no change in PFS.
				UWT excluded			
							OVWT *vs* NW: improved OS; no change in PFS;
Wang (25)	Mixed (lung 22.0%, melanoma 13.6%, ovarian 8%, other 52%)	250	NR	BMI: OB *vs* UWT+ NW+ OVWT	anti-PD-1 or anti-PD-L1	age, sex, ECOG status, line of treatment, cancer type	OB: improved OS and PFS
Cortellini (39)	Mixed (65% NSCLC, 18.7% melanoma, 13.8% RCC, 2.4% other	976	17 center retrospective study	BMI: OVWT+ OB *vs* NW+UW	anti-PD-1 or anti-PD-L1	primary tumor type, sex, age, ECOG, treatment line, # metastatic sites	High BMI (OVWT+OB): improved PFS.
							OB *vs* NW: improved OS but not PFS. OVWT *vs* NW: improved PFS and OS.
							NW/UW patients: 25.2% had irAEs; OVWT/OB patients had 55.6% irAEs
An meta-analysis (40)	Mixed (68.2% NSCLC; 18.5% melanoma, 10.2% RCC)	5279	Mix of trials and retrospective	BMI: NW+UWT *vs* OVWT+OB	anti-PD-1, anti-PD-L1, anti-CTLA-4	NR	High BMI (OVWT+OB): improved OS and PFS; no difference in irAEs

For studies based upon the use of BMI as the obesity defining metric, UWT, underweight (BMI<18.5 kg/m^2^); NW, normal weight (BMI 18.5-24.9 kg/m^2^); OVWT, overweight (BMI 25-29.9 kg/m^2^); OB, obesity (BMI > 30 kg/m^2^). Note that non-significant data trends are indicated as such; all other outcomes listed are significant as per the original report. AJCC, American Joint Committee on Staging; CRP, C reactive protein; ECOG, Eastern Cooperative Oncology Group performance status; ICI, immune checkpoint inhibitor; irAEs, immune-related adverse events; LDH, lactate dehydrogenase; NLR, neutrophil to lymphocyte ratio; not sig, not significant; NR, not reported; NSCLC, non-small cell lung carcinoma; OS, overall survival; PFS, progression-free survival; RCC, renal cell carcinoma; SOC, standard of care; Tx, treatment.

**Table 2:** Studies refuting the obesity paradox for cancer immunotherapy outcomes.

First author (Year)	Tumor type	Cohort size	Trial name or SOC	Obesity metric used	ICI used	Controlled prognostic factors	Outcomes
McQuade (34)	melanoma	117 females	retrospective analyses from 4 US and Australian centers	BMI: per WHO for NW, OVWT, OB. UWT excluded	anti-PD-1, anti-PD-L1, or anti-CTLA-4 + dacarbazine (chemotherapy)	age, AJCC & disease stage, LDH status, ECOG status	OB *vs* NW: trending worse PFS and no change in OS
Donnelly (37)	melanoma	423	prospective NYU Interdisciplinary Melanoma Cooperative Group	BMI: per WHO standards for UW, NW, OVWT, OB	anti-PD-1, anti-CTLA-4, or combination	1st *versus* 2nd+ line of therapy; age, gender, tumor stage, LDH, ECOG, # metastatic sites; mono *vs* combo therapy	All patients: OVWT and OB: no changes in PFS or OS. OVWT and OB: 2nd line+ ICI trended toward worse PFS and OS.
							OVWT and OB: trend toward worse PFS with anti-PD-1 alone
Rutkowski (41)	melanoma	688	retrospective; SOC 3 Italian and 2 Polish centers	BMI: per WHO standards for UW, NW, OVWT, OB	mixed ICIs	age, sex, first line ICI *vs* ICI sequencing, tumor mutation status, LDH, ECOG	BMI: no effect on PFS or OS for either 1st line ICI or ICI sequencing
Young (42)	Melanoma	287	Retrospective; SOC	BMI: NW, OVWT, OB or CT scan for TATI, SMD, SMG	mixed: anti-PD-1, anti-PD-L1 or anti-PD-1 + anti-CTLA-4	age, sex, stage, prior therapies	BMI: no effect on PFS or OS in total cohort or men or women analyzed separately.
							High TATI: worse PFS with no change in OS in total cohort.
							SMG: no effect on PFS or OS.
Di Filippo (43)	Melanoma	1214	French 26-center MelBase NCT02828202	BMI: per WHO standards for NW, OVWT, OB.	anti-PD-1 or anti-PD-1+anti-CTLA-4	Age, sex, ECOG, LDH, brain	BMI: no effect on PFS or OS or TRAEs.
				UWT excluded		metastases, tumor mutation status, # metastatic sites	Overall response rates did not differ across BMI categories.
Bergerot (44)	RCC	42	Retrospective	BMI: OVWT+OB *vs* NW+UW	anti-PD-1 or anti-PD-L1 or anti-PD-1+anti-CTLA-4	sex, race, IMDC score, histology	High BMI (OVWT +OB): trending worse OS (not sig.)
De Giorgi (45)	RCC	313	Italian Expanded Access Program	BMI: OVWT+ OB *vs* NW+UWT	only anti-PD-1 (nivolumab)	age, ECOG SII, NLR, PLR	NW+UWT: patients with SII ≥ 1375 had worse OS *vs* patients with low BMI + low SII or high BMI + low SII or high BMI + high SII
Boi (18)	RCC	72	Retrospective SOC only (2 US institutions)	BMI: OB *vs* OVWT+ NW.	anti-PD-1	age, sex, IMDC score, # prior therapies	OB *vs* OVWT+NW: decreased PFS and OS.
				UWT excluded			OVWT+OB *vs* NW: no change in PFS or OS
Khojandi (30)	Mixed tumor types	149	Retrospective SOC	BMI: OB *vs* OVWT+NW+UWT	anti-PD-1/PD-L1	sex	OB: no change in OS

For studies based upon the use of BMI as the obesity defining metric, UWT, underweight (BMI<18.5 kg/m^2^); NW, normal weight (BMI 18.5-24.9 kg/m^2^); OVWT, overweight (BMI 25-29.9 kg/m^2^); OB, obesity (BMI > 30 kg/m^2^). Note that non-significant data trends are indicated as such; all other outcomes listed are significant as per the original report. AJCC, American Joint Committee on Staging; CRP, C reactive protein; CT, computed tomography; ECOG, Eastern Cooperative Oncology Group performance status; irAEs, immune-related adverse events; LDH, lactate dehydrogenase; NLR, neutrophil to lymphocyte ratio; NR, not reported; NSCLC, non-small cell lung carcinoma; OS, overall survival; PFS, progression-free survival; PLR; platelet to lymphocyte ratio; RCC, renal cell carcinoma; SII, systemic inflammation index; SMD, smooth muscle density; SMG, smooth muscle gauge; SOC, standard of care; TATI, total adipose tissue index; TRAEs, treatment-related adverse events.

Evidence supporting the obesity paradox has also been found in tumor types other than melanoma. For example, a study by Kichenadasse et al. examined whether BMI was associated with PFS, OS, or treatment-related adverse effects in patients with non-small cell lung cancer (NSCLC) (35). In a retrospective evaluation of four clinical trials, pooled analyses were used to determine uniformity of identified associations using an expanded cohort of patients on the PD-L1 inhibitor atezolizumab. This study found that OS was significantly improved in patients with obesity who had PD-L1+ tumors (HR = 0.36 [95% CI, 0.21-0.62] for patients within the highest PD-L1 expression category), but that OS was not influenced by BMI in PD-L1^-^ tumors (**Table 1**). Notably, the authors found that treatment-related adverse events were not impacted by patient BMI. In this study, males comprised 62% of the cohort overall and the benefits of obesity were seen for both women and men (P value for interaction = 0.76) (35), unlike the prior report by McQuade and colleagues (34). In yet another report, An et al. performed a meta-analysis of thirteen pooled studies of NSCLC, melanoma, renal cancer, and other types of cancer patients treated with any of the following ICI agents: anti-PD-1 (pembrolizumab or nivolumab) or anti-PD-L1 (atezolizumab). Their analyses showed that OS and PFS were significantly improved for patients with high BMI *versus* patients with low BMI, as illustrated by significantly lower overall HRs for OS (HR = 0.62 [95% CI, 0.55-0.71] and PFS (HR = 0.71 [95% CI, 0.61-0.83]) (40) (**Table 1**). In this study, outcomes were pooled for males and females. Lastly, a retrospective study on 250 patients of mixed tumor types treated with anti-PD-1 or anti-PD-L1 determined that obesity improved both PFS (237 days *vs* 141 days, HR= 0.61 [95% CI, 0.42-0.89] and OS (523 days *versus* 361 days, HR of 0.59 [95% CI, 0.35-0.99]) after controlling for ECOG performance status, line of therapy, age, biological sex, and cancer type (25) (**Table 1**). Collectively, these reports provided strong evidence for the obesity paradox, giving rise to the widespread idea that obesity is typically associated with improved ICI outcomes in cancer patients, regardless of the type of cancer or ICI used.

#### Key Features of Immune Responses Vary Across Tumor Types

In considering the effects of obesity on immune responses to solid tumors and ICI outcomes, we propose that it is necessary to begin by evaluating whether fundamental differences exist in the nature of immune responses between cancer types. Evidence in support of this idea comes from a 2019 study by Thorsson et al., who published a detailed transcriptomic analysis of 33 distinct cancer types based upon analyses of over 10,000 different TCGA tumor samples (46). The authors identified six main categories of immune responses across tumor types, illustrating that baseline immune responses can vary widely from one tumor type to another. For example, in clear cell RCC, the most prevalent subtype of kidney cancer, the predominant immune response was one described as “inflammatory”, meaning that the ratios of macrophages to lymphocytes were balanced, the Th1 to Th2 ratio was high, and Th17 cells were elevated. In contrast, cutaneous melanoma tumors were dominated by two signatures: “wound healing”, characterized by a robust angiogenesis and proliferation in combination with a Th2 profile, and “IFN*γ* dominant”, characterized by high numbers of CD8+ T cells and a strong M1 to M2 ratio (46). Even when phenotypically similar leukocytes are present across tumor types, their functional status may differ, as in the finding that although PD-1^high^CD8+ T cells infiltrating melanoma tumors show severe functional exhaustion, phenotypically similar T cells from breast tumors retain polyfunctionality (47). Furthermore, as mentioned previously, a recent evaluation of human clear cell RCC biospecimens found that tumors with decreased expression of the metabolic enzyme PHD3 were significantly more likely to have an immunologically “cold” phenotype, wherein CD8+ effector T cell gene signatures were lacking (24). In contrast, the melanoma tumors examined did not display this relationship. Finally, our analysis of CD8+ TILs from renal cancer patients showed that obesity is associated with reduced frequencies of PD-1^high^CD8+ TILs (18), which differs from the lack of an association between obesity and PD-1+CD8+ TILs found in a combined cohort of melanoma and breast cancer patients (30). Collectively, these findings illustrate that immune responses to solid tumors can vary greatly between tumor types. It is therefore implausible that a single paradigm should be expected to accurately explain all interactions between host obesity, which is known to be heterogeneous in terms of the amount of inflammation and metabolic dysfunction present, and anti-tumor immunity, which is also known to vary across tumor types.

#### Is Renal Cancer an Obesity Paradox Outlier?

Given the fundamental differences present in the immune responses to renal cancer *versus* melanoma – or even among patients with renal tumors - it is perhaps not surprising that the reported effects of obesity on ICI outcomes in renal cancer patients are inconsistent. One of the first studies to report findings supporting an obesity paradox in renal cancer was the 2016 study by Albiges et al., which used a BMI cut-point of 25 kg/m^2^ (combining both overweight and obesity into one high BMI category) to examine TKI therapy outcomes (48). The authors found that in two separate cohorts of > 6000 RCC patients, high BMI was associated with improved OS; the authors then linked the protective effects of increased adiposity to decreased expression of the enzyme fatty acid synthase, which has been described by others to have tumor-promoting effects (49). However, this study did not examine ICI outcomes.

Since that time, the effects of obesity on ICI outcomes in RCC have been examined in multiple studies. A 2020 study by Sanchez et al. reported that in a cohort of 129 RCC patients treated with anti-PD-1, anti-PD-L1, or anti-PD-1+anti-CTLA-4, obesity (BMI ≥ 30 kg/m^2^) tended to improve OS (49.9 months with obesity *versus* 15.5 months without) (HR 0.54; 95% CI [0.31-0.95]), although this association was not significant after controlling for International Metastatic RCC Database Consortium (IMDC) risk score (HR = 0.72; 95% CI [0.40-1.30]) (32) (**Table 1**). Risk score consideration is important, as this metric is used to predict survival in RCC patients (*i.e.*, higher risk scores associate with worse survival). Notably, in this same study, the authors found that RCC patients treated with TKIs as part of the COMPARZ trial did, in fact, display significantly improved OS with obesity (median survival for RCC patients with obesity 35.7 months [27.7 months – not reached]; median survival for normal weight RCC patients 19.1 months [15.3-27.8], reflecting the earlier findings of Albiges et al. (48) Therefore, the same analytical approach yielded divergent obesity-related outcomes in patients treated with ICI *versus* TKI.

The Sanchez study is also important because it is one of the few studies to try to identify biological drivers of obesity-associated outcomes to ICI in humans. As mentioned earlier, the authors performed unbiased transcriptomic analyses of renal tumor tissue from the COMPARZ trial and from treatment-naive TCGA donors; surprisingly few immune-related changes were detected, and none were consistent between the two cohorts. Thus, it is possible that TKI treatment induced changes in the renal tumor microenvironment that are not present in treatment-naive tissues. Unfortunately, a similar transcriptomic analysis could not be conducted on tissues from the authors’ ICI cohort, and flow cytometric evaluation on a limited number of renal tumor specimens obtained from nephrectomy patients displayed no obesity-related changes in the frequency of major leukocyte populations examined (32). In this study, deeper phenotypic analyses of leukocyte subpopulations and activation or exhaustion states were not evaluated by flow cytometry, creating the possibility that subtle obesity-related distinctions actually do exist.

Two other studies have examined the effects of obesity on ICI outcomes in renal cancer. One, by Labadie et al., categorized their 90 RCC subjects by either exhibiting primary response to ICI (*i.e.*, patients who had received at least one dose of anti-PD-1 or anti-PD-L1 [pembrolizumab, nivolumab, atezolizumab]) or primary resistance to ICI. In patients who responded to ICI, improved PFS was associated with increasing BMI (p = 0.007) (**Table 1**) and the presence of immune-related adverse events (irAEs) (38). In these same patients, improved OS was significantly associated with overweight (BMI of 25 kg/m^2^ - 29.9 kg/m2; p = 0.03) but not obesity (BMI ≥30 kg/m^2^; p = 0.07). The authors went on to determine that the main drivers of primary resistance to ICI were worse Eastern Cooperative Oncology Group (ECOG) performance status at treatment initiation, earlier cancer stage, no prior nephrectomy, and no development of irAEs. Notably, BMI did not differ between the two groups, suggesting that this metric was not a major factor in determining response *versus* lack of response to ICI in this patient cohort (38). This study is noteworthy because it illustrates the complex interactions between obesity, overweight, and ICI outcomes. More recently, we analyzed a cohort of 72 RCC patients from two institutions who had received only anti-PD-1 (nivolumab or pembrolizumab) as standard of care (18). Using a BMI of 30 kg/m^2^ as the cut-point, we found that obesity was significantly associated with worse PFS and OS in our cohort; this resulted in a median PFS of 7.3 months for patients with obesity *versus* 13.8 months for those without (p = 0.0322) and a median OS of 18.0 months for patients with obesity *versus* 30.0 months for those without (p = 0.0371). After controlling for IMDC risk score, age, and biological sex, obesity was also associated with an increased risk of death (*i.e.*, the HR for patients with a BMI < 30kg/m^2^ = 0.48; 95% CI [0.24-0.96]) (18) (**Table 2**). Collectively, these three studies demonstrate that obesity-associated outcomes can vary greatly in RCC patients treated with ICIs, ranging from significantly worse survival to a trending increase in improved survival. This variability implies that underlying cohort demographic differences and/or alterations in study methodology are important contributing factors to identified outcomes.

Several other studies have examined associations between increasing adiposity (BMI ≥25 kg/m^2^) and ICI outcomes in RCC. This is a critical distinction, because such studies combine evaluations of overweight plus obesity on ICI outcomes, rather than obesity alone. In our RCC cohort, when a BMI cut-point of ≥25 kg/m^2^ was used instead of ≥30 kg/m^2^, we found that the curves for PFS and OS were nearly overlapping (18), demonstrating the beneficial effects of overweight *versus* obesity in our patient cohort and reflecting trends identified in the Labadie study (38). In another recent study, increased adiposity was found to produce disparate outcomes between TKI- treated and ICI-treated patients, reminiscent of the Sanchez findings. Using a cohort of 42 RCC patients treated with anti-PD-1 or anti-PD-L1 (nivolumab, atezolizumab, avelumab), Bergerot et al. reported that combined overweight plus obesity had a trending but non-significant detrimental effect on survival (19.9 months for overweight plus obesity *vs* 23.6 months in normal weight, p = 0.26) (44) (**Table 2**). At the same institution, obesity was associated with significantly improved outcomes in RCC patients treated with TKIs (OS = 36 months for high BMI *versus* 24 months for low BMI (p = 0.02), illustrating divergent effects of increasing BMI on ICI *versus* TKI efficacy (44). However, in other studies, a BMI ≥25 kg/m^2^ was found to be protective in RCC patients treated with ICIs (39, 45). Of these studies, one by de Giorgi et al. evaluated outcomes following only anti-PD-1 (nivolumab) in a cohort of 313 RCC patients who had progressed on VEGF- targeting therapy (45), whereas the other, by Cortellini et al., evaluated outcomes following anti-PD-1 or anti-PD-L1 (pembrolizumab, nivolumab, atezolizumab) in a mixed cohort of 976 cancer patients, of whom 135 had RCC (39). The Cortellini study found that RCC patients with overweight or obesity experienced longer survival than did patients of normal weight (HR = 0.61; 95% CI [0.45-0.80], p = 0.0005) (**Table 1**). The de Giorgi study is notable because the authors also examined the combined effects of increasing BMI and systemic inflammation on OS after ICI administration. Here, systemic inflammation was defined by multiplying the baseline peripheral blood platelet (P) value and the neutrophil to lymphocyte (N/L) ratio [thus, the systemic inflammation index = P x N/L]. This analysis revealed that individuals with low inflammation had better OS, a finding that held true for patients with either high or low BMI status. Patients who displayed low systemic inflammation plus high BMI (≥25 kg/m^2^) had the best outcomes, followed by patients with low inflammation and low BMI (<25 kg/m^2^), then patients with high systemic inflammation plus a high BMI; those individuals with high systemic inflammation plus a low BMI (<25 kg/m^2^) experienced the worst OS (45) (**Table 2**). These findings suggest that low inflammation, and not BMI status, may be a primary determinant of improved survival after ICI therapy in RCC patients. The contributions of systemic inflammation to ICI outcomes warrants further investigation, particularly as it provides a human correlate to pre-clinical findings that elevated concentrations of the inflammatory mediator IL-1b within renal tumors contributed to immunotherapy resistance in mice with DIO (18).

### Revisiting the Obesity Paradox in Cancer Immunotherapy

#### Evidence Against the Paradigm in Melanoma

As detailed above, the conflicting body of evidence regarding the existence of an obesity paradox in the context of cancer immunotherapy implies that this paradox should not be generalized across all cancer types and patient populations. Even in metastatic melanoma where the paradox was first described in patients receiving ICIs by McQuade et al., the association between obesity and improved PFS and OS was only seen in male patients in two immunotherapy cohorts (one randomized controlled trial of ipilimumab plus the chemotherapeutic agent dacarbazine, and a retrospective cohort treated with pembrolizumab, nivolumab, or atezolizumab) (34). The same report found no significant association, either positive or negative, between obesity and ICI outcomes in female melanoma patients (34). More recent studies have found that in metastatic melanoma patients receiving ICIs, patients who had overweight or obesity did not have different PFS than patients with normal BMI (37, 42). Indeed, an association with better PFS in patients with overweight or obesity was only observed by Donnelly et al. after stratifying their cohort of 423 metastatic melanoma patients by first line *versus* non-first line ICI recipients (37). Thus, in patients who received first line ICIs (n=272), both overweight and obesity tended to improve PFS (p = 0.17) and OS (p = 0.47) (**Table 1**), although neither association reached significance. In contrast, for patients who did not receive ICIs as their first line of therapy, those with overweight and obesity trended toward worse PFS (p = 0.51) and OS (p = 0.42) (**Table 2**). Furthermore, only patients with overweight or obesity who received combination ICIs (*i.e.*, anti-CTLA-4 + anti-PD-1) had a statistically significant improvement in PFS (p = 0.0044) and trended toward improved OS (p = 0.47). Patients with overweight or obesity who received only anti-PD-1 showed a trend toward worse PFS (p = 0.40) but no clear change in OS (p = 0.35) (37). In addition, a 2020 study by Young et al. found no associations – either positive or negative –between BMI and ICI outcomes in a cohort of 287 men and women with melanoma (42). These authors found that by assessing body composition in a more nuanced manner using computed tomography (CT) scan-based data, rather than BMI, individuals with a high total adiposity index (i.e. subcutaneous adipose tissue area + visceral adipose tissue area/height^2^) experienced worse PFS following ICI (HR for PFS = 1.71; 95% CI [1.01-2.87]; p = 0.04) (**Table 2**). This relationship was particularly strong for women (HR for PFS = 2.06; 95% CI [1.06-3.98]; p = 0.032) but not men (HR for PFS = 1.40; 95% CI [0.59-3.31]; p = 0.45) (39). The 2021 study by Khojandi et al. found that in a cohort of 129 melanoma patients treated with anti-CTLA-4 (ipilimumab) a BMI ≥ 30kg/m^2^ at treatment initiation was associated with better OS (p = 0.0368) (**Table 1**) but had no effect on PFS (30). However, the same authors found that in a cohort of 149 patients with melanoma or other tumor types who were treated with anti-PD-1 (nivolumab or pembrolizumab) or anti-PD-L1 (atezolizumab or durvalumab), obesity defined by BMI had no effect on OS (p = 0.7003) (**Table 2**), reflecting the findings of Young et al. (42) In looking further at these disparate results, Khojandi et al. found that the relative amount of oxidized low density lipoproteins (ox-LDL) in circulation predicted anti-PD-1 or PD-L1 outcomes, with high ox-LDL being associated with poor OS, due to the ability of ox-LDL to activate the cytoprotective molecule hemeoxygenase -1 (HO-1) in tumor cells, which made them less susceptible to T cell-mediated killing (30). In support of the above findings that counter the obesity paradox paradigm, Rutkowski et al. reported that BMI had no effect on OS in a cohort of 688 metastatic melanoma patients receiving first line ICI (HR = 1.02; 95% CI [0.99-1.05]; p = 0.202) or ICI sequencing (HR 1.02; 95% CI [0.99-1.04]; p = 0.237) in Italy and Poland (41) (**Table 2**) and Di Filippo et al. found that BMI status did not predict PFS (p = 0.88) or OS (p = 0.25) in a cohort of 1214 melanoma patients treated with first line ICI or targeted therapies in the French 26-center prospective MelBase study (43) (**Table 2**). Thus, even for patients with the same tumor type – melanoma - divergent associations between obesity and ICI outcomes have been reported. The reasons underlying these findings that contradict the obesity paradox paradigm must be interrogated further, as doing so could yield a fuller understanding of the biological drivers of obesity-associated ICI outcomes in cancer patients. In the following sections, we discuss several factors that could be critical contributors to the divergent findings reported to date regarding the effects of obesity on ICI efficacy.

#### The Wide Range of ICIs Used in RCC Studies

With regard to RCC specifically, one notable difference among the RCC studies summarized above is the fact that all but two examined patient outcomes while combining multiple types of administered ICIs. The de Giorgi study that reported on improved ICI outcomes with low inflammation analyzed data from patients treated only with anti-PD-1 (45). The dates of treatment range from July 2015 - April of 2016, and these patients were treated in Europe. Our group’s Boi et al. study examined outcomes only in RCC patients who received anti-PD-1 monotherapy as standard of care in the U.S. between December 2015 - July of 2019 (18). All other studies have combined analyses for anti-PD-1 and anti-PD-L1 monotherapies or included combinatorial anti-PD-1 + anti-CTLA-4. Therefore, both the de Giorgi and Boi et al. study designs contrast with other studies, which performed analyses across multiple immunotherapeutic agents, and/or a combination of clinical trial and standard of care ICI administration (25, 32, 34, 39). It is thus conceivable that no clear consensus regarding the effects of obesity on immune checkpoint inhibitor outcomes in RCC has yet emerged because of the dissimilar nature of the analyses performed. Although the Donnelly et al. study analyzed data from melanoma patients, their results clearly illustrated that monotherapy ICI (ex: anti-PD-1) can lead to very different outcomes *versus* combination ICIs (anti-PD-1 + anti-CTLA-4) (37).

#### The Case for Re-Examining ICI Dosing Regimens as Related to the Obesity Paradox

Aside from methodological differences in treatment approaches, there are multiple other factors underlying the complex relationship between obesity and response to ICIs that may contribute to the observed discrepancies in reported outcomes. One factor that is a potential confounder of the relationship between BMI and ICI responses is the ICI dose administered. Currently, most ICIs (nivolumab, pembrolizumab and atezolizumab) are administered to patients during standard of care practice as a flat dose (*i.e.* a fixed dose rather than a body-surface area or weight-based dose) while others (including ipilimumab, avelumab and durvalumab) are weight-based (50). Of note is the fact that nivolumab was initially administered at a 3 mg/kg dose, but was changed to a flat dose of 240 mg every two weeks in 2016 (https://www.fda.gov/drugs/resources-information-approved-drugs/modification-dosage-regimen-nivolumab). For a cancer patient of 80 kg (176 pounds), dosing under the two regimens would be equivalent, but for patients at the upper and lower ranges of body weight, administered antibody amounts would be vastly different for flat *versus* escalated dosing. This raises the possibility that retrospective studies on obesity and ICI outcomes may exhibit different trends depending on what percentage of patients began treatment with flat *versus* escalated dosing. Such detailed information is typically not published in retrospective outcome studies but performing retrospective analyses of this type may provide insight into reported discrepancies in obesity-related ICI outcomes. Notably, flat doses are recommended for drugs whose pharmacokinetics are not significantly affected by body weight, as well as drugs with flat exposure–response relationships, whereby variations in exposure do not affect clinical outcome. Studies have shown that weight-based doses and fixed doses are comparable in exposure, safety and efficacy for nivolumab and pembrolizumab (50, 51) but not ipilimumab (52). However, the extent to which ICI dosing has impacted reported connections between obesity and therapeutic outcomes is not currently known.

#### The Impact of Cachexia on ICI Outcomes

Another potential confounder that can result in suboptimal ICI efficacy is cachexia, defined as a cancer-associated loss in weight (specifically, a loss in skeletal muscle mass, also referred to as sarcopenia) that is accompanied by metabolic wasting (53). Although cachexia-associated weight loss can decrease BMI scores, it is nonetheless associated with poor responses to ICI [reviewed in (54)], which may explain why individuals with lower body mass are frequently observed to have worse ICI outcomes. Cancer-associated cachexia is promoted by chronic inflammation mediated through several proinflammatory cytokines, including interleukin-1 (IL-1), IL-6, and tumor necrosis factor alpha (TNF-α) (55, 56). Patients receiving ICI may be particularly susceptible to cancer-associated cachexia, and the resulting immunosuppressive environment may lead to primary resistance to immunotherapy (57). The link between sarcopenia and ICI outcomes was recently studied by Chu et al. in a cohort of 97 metastatic melanoma patients treated with anti-CTLA-4 (ipilimumab), wherein the authors found that low smooth muscle density resulted in worse PFS (2.4 months *vs* 2.7 months for high smooth muscle density; HR = 1.76, p = 0.008) and OS (5.4 months *versus* 17.5 months for high smooth muscle density; HR = 2.47, p =0.001) (58). In contrast, high smooth muscle density was associated with a greater rate of irAEs and lower baseline neutrophil to lymphocyte ratios in peripheral blood (58). These findings are notable for several reasons: first, Labadie et al. reported that RCC patients who experienced irAEs also tended to experience a positive, primary response to administered ICIs (38); second, de Giorgi et al. found that high systemic inflammation, defined in part by using the neutrophil to lymphocyte ratio, was linked to worse ICI outcomes (45). The Chu results therefore suggest that retained muscle mass in cancer patients is linked to both more robust immune responses and decreased inflammation. At present, connections between sarcopenia or cachexia and the quality of protective anti-tumor immunity are unclear. However, it is possible that in the de Giorgi study, their findings that high inflammation was associated with worse ICI outcomes (45) may have been due to the presence of cachexia in these patients – a condition that would not have been detected by the use of BMI. In another retrospective analysis of from two randomized KEYNOTE trials of anti-PD-1 (pembrolizumab) in melanoma and NSCLC patients (n=1144), decreased OS was observed in patients with higher pembrolizumab baseline clearance (melanoma HR = 2.56; 95% CI [1.72-3.80] and NSCLC HR = 2.64; 95% CI [1.94-3.57]), a finding that was positively associated with markers of cachexia (59). Finally, the previously mentioned 2020 study by Young et al. demonstrated that males and females with low skeletal muscle gauge, an index that suggests skeletal muscle loss consistent with sarcopenia, plus high total adiposity experienced the poorest PFS (p = 0.021) and OS (p = 0.021) in their cohort of melanoma patients treated with ICIs (42). Thus, we propose that longitudinal weight and skeletal muscle mass measurements, the latter as determined by CT scan, should be routinely implemented in future studies to permit the identification of not only cachexia at treatment onset but cachexia development during ICI treatment. Doing so may reveal important associations between body composition flux and ICI outcomes that are missed when a single static BMI measurement at treatment initiation is used to define obesity.

#### Limitations of Using BMI as the Obesity-Defining Metric

A third confounding factor verges on using BMI as a readout for obesity. Although widely used, BMI does not reflect the proportions of muscle and fat or their distribution (intra-abdominal *vs.* subcutaneous), which are further influenced by sex and ethnicity (7). A BMI range of 25-35 kg/m^2^ (overweight and grade I obesity) can also potentially include lean patients with high muscle mass, such as athletes (60). On the other hand, patients with abnormal fat distribution (excessive intra-abdominal or visceral fat) may have normal or slightly overweight BMI scores (61). Excessive visceral fat in obesity predisposes to the metabolic syndrome that is associated with insulin resistance and cardiovascular disease. Yet, it has been estimated that about 10% of adults in the U.S. have a BMI value that categorizes them as having obesity while also maintaining a healthy metabolic status (normal insulin sensitivity and low risk of cardiovascular disease), compared with 8% of adults who have a normal BMI and are metabolically unhealthy (62). Indeed, a recent study of pro-inflammatory markers in the plasma of volunteers with Grade II or higher obesity (BMI ≥ 35 kg/m^2^) found considerable person-to-person variation, ranging from markedly elevated to normal concentrations of many parameters measured, including IL-6 and C-reactive protein (CRP) (29). Thus, BMI often does not accurately reflect an individual’s metabolic status, which may have confounding and dire consequences on patient responses to ICIs. It is for these reasons that Caan et al. have proposed that a “BMI paradox”, rather than a true obesity paradox, exists in cancer patient outcomes, due to the imprecise nature of BMI as a tool to define actual obesity (63). In one study, the Caan group found that sarcopenia was associated with a 27% increase in mortality *versus* colorectal cancer patients who did not have sarcopenia; notably, the authors found that patients with the lowest overall risk of mortality had a BMI between 25 – 29.9 kg/m^2^ (i.e. they were overweight) and also had the lowest prevalence of sarcopenia (64).

Non-BMI measures of adiposity include waist circumference, which is a readout of central adiposity and has been shown to be a stronger predictor of all-cancer risk than BMI (65). Previously, we reported that waist circumference, but not BMI, was associated with increased renal tumor complexity, which is a metric used to evaluate cancer aggressiveness (66). Thus, fat distribution may be particularly important as an influence of outcomes in renal cancer. Other adiposity measures include the intermuscular fat index (IFI), subcutaneous fat index (SFI), visceral fat index (VFI), perinephric fat thickness (PNF), visceral-to-subcutaneous fat area ratio (V/S) (67), and total adiposity index (SFI + VFI), all of which attempt to capture the amount of fat and/or its distribution as measured from CT scans. Notably, several studies have shown that subcutaneous fat is associated with a reduction in cancer mortality risk, a beneficial association not seen with intermuscular or visceral fat (63, 68). Evaluations of fat distribution have been employed in recent studies to assess potential correlations between adiposity and cancer therapy outcomes, including immunotherapy (67, 69). Clark et al. compared two visceral adiposity measures (perinephric fat [PNF] and the ratio of visceral to subcutaneous fat [V/S]) with BMI in predicting outcome among patients undergoing neoadjuvant chemoradiation and resection for locally advanced rectal cancers (68). They found that obesity, as defined by elevated V/S or PNF but not BMI, was associated with shorter disease-free survival (p = 0.02) and OS (p = 0.047). Grignol et al. used V/S as a visceral adiposity metric and found that increased V/S, but not BMI, was associated with decreased PFS (p=0.009) and OS (p=007) in patients with metastatic melanoma treated with bevacizumab ± IFN-α (67). It is thus possible that such precise measures of body fat distribution and muscle composition may present a clearer picture of the underlying associations between adiposity and clinical ICI outcomes.

## Discussion

The questions of whether and how obesity affects cancer immunotherapy outcomes are critical ones, particularly given the steadily rising prevalence of obesity in adults worldwide. Although the amount of research being dedicated to these questions is growing rapidly, investigators and clinicians still lack a full, nuanced understanding of when obesity is beneficial for ICI outcomes and when it is not. In addition, the mechanisms underlying both positive and negative obesity-associated cancer ICI outcomes remain poorly understood. Thus, there is much work to be done in this area. We caution that an over-reliance on the obesity paradox paradigm may hinder urgently needed progress, as it may lead to an unwarranted disregard of studies that have shown opposing outcomes.

Obesity is a heterogeneous disease characterized by considerable fluctuations in fat distribution, inflammation, muscle density, and insulin resistance, among others. The contributions of these factors to cancer patient outcomes following ICI therapy are far from clear. As mentioned above, several lines of evidence point to the fact that systemic or intra-tumoral inflammation may be particularly important in determining ICI success or failure – independently of BMI status. In addition, more precise measures of adiposity and fat distribution, such as those based upon CT scan analyses rather than BMI, should lead to more informative and accurate insight into the effects of host obesity on ICI outcomes. Although BMI is an easily calculated metric, we highlighted multiple studies that illustrate its shortcomings, as others have done before us. Unfortunately, for many retrospective analyses – including our own – static BMI calculations at treatment initiation are the most readily available method of evaluating obesity within patient cohorts. Prospective studies aimed at examining changes in body composition over the course of ICI administration would be more challenging to perform but would provide a vastly deeper understanding of how adiposity and fat deposition impact ICI efficacy and patient outcomes. At the same time, future studies should try to incorporate assessments of not just adiposity, but also inflammation and muscle density, as ample evidence already exists to demonstrate that these factors influence ICI outcomes. Future studies should build upon the work of de Giorgi et al. (45) and Young et al. (42), for example, to continue to deepen our understanding of how the interplay between multiple factors (obesity and inflammation, or obesity and muscle density, respectively) contributes to ICI efficacy. In addition, other obesity-associated physiologic and metabolic alterations should be explored so needed insight is gained regarding the coordinate impact of obesity plus insulin resistance or obesity-associated microbiota alterations on cancer patient outcomes following ICI therapy administration. In the meantime, the variability in patient outcomes that have been described while using BMI to define obesity should not be overlooked. Studies such as that by McQuade et al. (34) indicate that closer attention should be paid to differences in response between males and females, as the causes of the divergent outcomes reported by these authors are not yet understood and could be the result of factors such as fat deposition or inflammation, in addition to the more obvious hormonal differences.

It is now clear from multiple lines of evidence that immune responses to solid tumors are also heterogeneous; they differ across solid tumor types and even across sub-regions within the same tumor mass. Consistent themes in pre-clinical murine modeling of obesity include metabolic perturbations in cytolytic cells, decreased CD8+ TIL effector function, and increased intra-tumoral suppressive myeloid signatures. However, the effects of obesity on anti-tumor immunity in cancer patients are much less clear and show variability even between patient cohorts with the same type of cancer, analyzed by the same researchers, as evidenced by the work of Sanchez et al. (32) Currently, many of the obesity-associated alterations in human and murine tumor immunity that have been identified suggest poorer baseline immunity that would portend an increased likelihood of treatment failure. How then do these studies of impaired tumor immunity coincide with patient outcome data illustrating that obesity improves ICI outcomes in so many patients? Wang et al. proposed that obesity-associated increases in PD-1 expression on CD8+ TILs promoted heightened anti-PD-1 efficacy (25), and this seems plausible in cases where such a positive association occurs. However, in cases where obesity is either not associated with higher PD-1 expression (30) or is associated with lower PD-1 expression (18), we should therefore expect that anti-PD-1-based therapies would be less effective and this has indeed been observed (18, 30). In addition, intriguing results from Donnelly et al. illustrate that even within the same cohort of melanoma patients, obesity-associated outcomes diverge for anti-PD-1 monotherapy *versus* anti-PD-1 + anti-CTLA-4 combination therapy (37); this finding suggests that underlying therapy-induced immune responses may be differentially impacted by host obesity. Indeed, both anti-PD-1 and anti-CTLA-4 are more effective in tumors that are infiltrated by T cells, but they act at different stages of the T cell-mediated immune response. Prior evidence shows that anti-CTLA-4 primarily improves T cell activation in tumor-draining lymph nodes by releasing inhibition mediated by CTLA-4-expressing antigen-presenting cells, but it can also enhance T cell effector function through effects on both exhausted CTLA-4-expressing TILs and CTLA-4^+^ regulatory T cells in the tumor (70, 71). Notably, the cellular targets of anti-PD-1 appear to be different. Initially, anti-PD-1 was thought to impact T cell effector function by releasing inhibition on exhausted PD-1+ CD8 TILs, *via* blocking interactions with PD-L1-expressing tumor cells or intratumoral leukocytes such as macrophages and MDSCs (72). However, more recent studies have shown that anti-PD-1 acts upon TCF1+CD8+ TILs that have stem-like properties and are capable of undergoing robust expansion following receptor ligation (73, 74). In addition, anti-PD-1 induces reinvigoration and expansion of exhausted CD8+ T cells in the peripheral blood (75). Combination anti-PD-1/anti-CTLA-4 therapy induces a unique immune response wherein activated but terminally differentiated CD8 TILs and T helper 1 (Th1) cells expanded (76). Finally, because PD-1 is expressed on myeloid cells, the use of anti-PD-1 may relieve immune suppression originating from this cell lineage (77). Thus, various ICIs target various immune cells, which may in turn be divergently impacted by obesity, ultimately leading to heterogenous outcomes. Clearly, much additional work remains to be done. Future studies should examine factors including the role of mast cells, macrophage polarization, TIL localization, and leukocyte metabolism, in both mice and humans receiving ICI therapies to deepen our insight regarding the ways in which obesity impacts anti-tumor immunity and ICI efficacy.

We urge continued and thoughtful investigation into this important area of study. Obesity is a complex and heterogeneous disease. Anti-tumor immunity is equally complex and variable. The number and type of ICI-based mono- and combinatorial therapies are expanding rapidly. Therefore, it makes little sense to attempt to force a “one-size-fits-all” paradigm to adequately describe the outcomes of myriad interactions between host obesity, anti-tumor immunity, tumor type, and therapeutic regimen. A closer interrogation of contrary results, and specifically the immune and metabolic profiles of patients who respond to therapy *versus* those who do not, could hold important keys to a fuller awareness of the biology that underpins obesity-associated cancer patient outcomes. Those keys are present, if only we will continue to look for them.

## Author Contributions

LN conceived of the review, helped to write and edit the review, and managed the project. AM helped to write and edit the review. KF helped to write the review. All authors contributed to the article and approved the submitted version.

## Funding

The UAB O'Neal Comprehensive Cancer Center (to LAN).

## Conflict of Interest

Author AM is employed by the company Adicet Bio.

The remaining authors declare that the research was conducted in the absence of any commercial or financial relationships that could be construed as a potential conflict of interest.

## Publisher’s Note

All claims expressed in this article are solely those of the authors and do not necessarily represent those of their affiliated organizations, or those of the publisher, the editors and the reviewers. Any product that may be evaluated in this article, or claim that may be made by its manufacturer, is not guaranteed or endorsed by the publisher.
